# A Prospective Cohort Study of Absconsion Incidents in Forensic Psychiatric Settings: Can We Identify Those at High-Risk?

**DOI:** 10.1371/journal.pone.0138819

**Published:** 2015-09-24

**Authors:** Alexis E. Cullen, Amelia Jewell, John Tully, Suzanne Coghlan, Kimberlie Dean, Tom Fahy

**Affiliations:** Department of Forensic and Neurodevelopmental Sciences, Institute of Psychiatry, Psychology & Neuroscience, King's College London, London, United Kingdom; Medical University of Vienna, AUSTRIA

## Abstract

**Background:**

Incidents of absconsion in forensic psychiatric units can have potentially serious consequences, yet surprisingly little is known about the characteristics of patients who abscond from these settings. The few previous studies conducted to date have employed retrospective designs, and no attempt has been made to develop an empirically-derived risk assessment scale. In this prospective study, we aimed to identify predictors of absconsion over a two-year period and investigate the feasibility of developing a brief risk assessment scale.

**Methods:**

The study examined a representative sample of 135 patients treated in forensic medium- and low-secure wards. At baseline, demographic, clinical, treatment-related, and offending/behavioural factors were ascertained from electronic medical records and the treating teams. Incidents of absconsion (i.e., failure to return from leave, incidents of escape, and absconding whilst on escorted leave) were assessed at a two-year follow-up. Logistic regression analyses were used to determine the strongest predictors of absconsion which were then weighted according to their ability to discriminate absconders and non-absconders. The predictive utility of a brief risk assessment scale based on these weighted items was evaluated using receiver operator characteristics (ROC).

**Results:**

During the two-year follow-up period, 27 patients (20%) absconded, accounting for 56 separate incidents. In multivariate analyses, four factors relating to offending and behaviour emerged as the strongest predictors of absconsion: history of sexual offending, previous absconsion, recent inpatient verbal aggression, and recent inpatient substance use. The weighted risk scale derived from these factors had moderate-to-good predictive accuracy (ROC area under the curve: 0.80; sensitivity: 067; specificity: 0.71), a high negative predictive value (0.91), but a low positive predictive value (0.34).

**Conclusion:**

Potentially-targetable recent behaviours, such as inpatient verbal aggression and substance use, are strong predictors of absconsion in forensic settings; the absence of these factors may enable clinical teams to identify unnecessarily restricted low-risk individuals.

## Introduction

Incidents of absconsion, defined as being absent from hospital without permission, are fortunately rare in forensic psychiatric settings [1]. However, these rare incidents can have potentially tragic outcomes including the occurrence of further incidents of serious violence; and during the past decade there have been several high profile cases of this nature in the UK. Such cases can lead to public inquires [2,3] and attract considerable media attention, both of which are likely to undermine the public’s confidence in the ability of these services to safely treat offenders with mental health problems. Furthermore, these cases may further increase stigma towards people with mental illness in the general population by enhancing the link between mental illness and risk of harm. In addition to the risk posed to members of the public by patients who abscond, it is important to emphasise the increased risk to the patients themselves. The UK National Confidential Inquiry recently reported that 25% of all psychiatric inpatient suicides occurred among those who had absconded from hospital [4]. Despite the potentially negative consequences associated with absconsion incidents, relatively little is known about the characteristics of patients who are most likely to abscond from forensic settings. The absence of rigorous research findings limits the ability of clinicians to make informed decisions about leave and how to manage the level of risk. As such, in an attempt to reduce the potential for high-impact events, clinicians may be unnecessarily restricting or denying leave to patients who are actually at low risk for absconsion events.

To date, nine studies (summarised in Table 1) have attempted to identify patient characteristics associated with absconsion from forensic psychiatric services. These studies have observed that several demographic factors, such as younger age, male sex, ethnicity, length of stay, and legal section are associated with absconsion [5,6,7,8,9,10]; however, findings are inconsistent across studies. With regards to diagnosis, perhaps unsurprisingly, psychopathy and personality disorder appear to be more common among those who abscond from forensic settings [7,11], a finding which is consistent with that of an early study which showed that absconders are characterised by higher scores on the Minnesota Multiphasic Personality Inventory than non-absconders [12]. In contrast, one reasonably consistent finding is that the presence of psychotic disorder is not associated with higher likelihood of absconsion [5,8,9,13]. A number of offending and behavioural factors have also been found be more common among patients who abscond relative to those who do not, including, a violent or acquisitive index offence [5,7,11], an admission precipitated by an index offence [6], a greater number of previous convictions [9], prior substance use problems [9,10,13], and higher scores on the Historical, Clinical, Risk-Management—20 (HCR-20 [14]) violence risk assessment tool [10]. Finally, several studies have reported that previous absconsion is associated with increased likelihood of later absconsion [5,6,10,13], indicating that past behaviour is a predictor of future behaviour, or at least that the factors associated with the past behaviour are still present and increase the risk of future behaviour.

**Table 1:** Studies examining characteristics of individuals who abscond from forensic settings.

Study	Setting (Country)	Design	Outcome	Factors associated with absconsion
Beer et al. (2009)]	Low secure inpatient unit (UK)	Case control (N = 17 vs. 61)	Absconsion, failure to return, escape	History of absconsion; history of substance misuse and dependence; history of non-compliance; history of sexually inappropriate behaviour; history of childhood conduct problems
Brook et al. (1999) [5]	Special hospital (UK)	Case control (N = 36 vs. 150)	Absconsion and escape	Younger age; shorter duration of stay; treated on medium-/high-dependency wards; previous absconsion; offending history; treatment non-compliance antagonism to hospital rules; impulsive/aggressive behaviour; deteriorated mental state; family/friends disagree with detention; history of acting out behaviour; anxiety conflict regarding transfer; judged at high-risk of absconsion
Cooke and Thorwarth (1978) [12]	Forensic psychiatric unit (US)	Case control (N = 30 vs. 30)	Escape	Minnesota Multiphasic Personality Inventory (MMPI [19]) scores
Dolan and Snowden (1994) [6]	Medium secure unit (UK)	Case control (N = 27 vs. 238)	Escape	Male gender; African-Caribbean ethnicity; legal status; referral from prison/police custody; offending prior to admission; history of absconsion
Gacono et al. (1997) [11]	Maximum security hospital (US)	Case-control (N = 18 vs. 18)	Escape	Malingering; non-psychotic disorder; not treated with antipsychotics; violent index offence; PCL-R [21] total, factor 1, and factor 2 scores
Huws and Shubsachs (1993) [7]	Special hospital (UK)	Case control (N = 66 vs. 7173)	Absconsion and failure to return	Shorter length of stay prior to absconsion; legal classification of psychopathy; legal classification of psychopathy; section 3 of MHA; property index offence
Moore and Hammond (2000) [8]	Special hospital (UK)	Case control (N = 44 vs. 5133)	Absconsion and failure to return	Younger age; personality disorder diagnosis; MHA section
Morrow (1969) [9]	Maximum security hospital (US)	Case control (N = 40 vs. 80)	Escape	Younger age; transfer from prison; non-psychotic disorder; number of previous convictions; unemployment; history of alcohol problems; sibling position
Wilkie et al. (2014) [10]	Forensic psychiatric hospital (Canada)	Case control (N = 57 vs. 57)	Absconsion and failure to return	Longer length of stay; history of unsuccessful absconsion attempts; co-morbid substance use disorder; higher HCR-20 [14] scores; fewer violent offences

Note. PCL-R: Psychopathy Checklist–Revised; MHA: Mental Health Act; HCR-20: Historical, Clinical, Risk-Management– 20

Whilst the aforementioned studies have helped to identify patient characteristics associated with absconsion, our current knowledge is limited by several methodological issues. Firstly, all studies conducted in forensic settings to-date have been retrospective in nature. This introduces the possibility that reverse causality may have contributed to some of these findings. For example, it is conceivable that patients who abscond may later receive a higher score on the HCR-20 as a result of this behaviour, rather than high HCR-20 scores being a true risk factor for absconsion. A second limitation relates to the fact that many studies have focused to a large extent on static, historical factors as opposed to dynamic factors. As such, it is not known if the risk of absconsion might be reduced by targeting risk factors that are potentially amenable to treatment. Finally, although previous studies have identified a range of factors that may increase the risk for absconsion, there has been no attempt to develop a statistically-derived risk assessment tool. This is surprising when one considers that there are now 25 different violence risk assessment scales in existence [15].

The current study examines data collected in a two-year follow-up of a sample of patients treated in medium- and low-secure forensic psychiatric services in the UK. The primary aim of the study was to advance existing knowledge by identifying patient characteristics prospectively associated with absconsion from forensic psychiatric units. Based on previous research, we examined a range of demographic, clinical, treatment-related, and offending/behavioural factors to determine those distinguishing patients who abscond from forensic settings from those who do not. An additional aim of the study was to investigate the feasibility of developing a brief absconsion risk assessment scale for use in forensic secure units.

## Methods

### Sample and setting

The current study was conducted within the South London and Maudsley (SLaM) National Health Service (NHS) Foundation Trust which provides secondary mental health care to individuals residing in four southeast London boroughs (Lambeth, Southwark, Lewisham, and Croydon). The sample comprised patients examined in a census conducted in November 2011, which included all individuals receiving treatment in SLaM forensic inpatient services within a two-week period. At the time of the census, SLaM forensic inpatient services were provided by two medium-secure units (eight medium-secure wards in total) and one low-secure unit consisting of a single ward. The medium-secure units provided a variety of services and included a psychiatric intensive care unit, a specialist personality disorder service, and a female only ward. In total, 135 patients were examined in the census.

### Data extraction

Census data were obtained using the Biomedical Research Centre (BRC) Clinical Records Interactive Search (CRIS) tool, which provides access to anonymised data derived from SLaM electronic medical records [16]. The CRIS tool has been described in detail previously [16,17,18]. In brief, CRIS facilitates the searching and retrieval of anonymised data from the medical records of over 165,000 patients who have been in contact with SLaM services from 2006 onwards (i.e., when electronic records were implemented across the trust). CRIS was approved as a dataset for secondary analysis by the Oxfordshire Research Ethics Committee C (08/H0606/71).

The aim of the census was to obtain detailed demographic, clinical, treatment-related, and offending/behavioural data for a representative sample of forensic inpatients and to follow this cohort longitudinally, thereby allowing us to identify predictors of adverse outcomes such as absconsion. A pilot study determined that several variables of interest, particularly those relating to offending and behaviour, were not systematically recorded within electronic medical records and could not be easily extracted using CRIS. We therefore developed a census form to capture additional variables of interest which were completed at the patient’s ward round with input from the multidisciplinary team. Census forms were then uploaded to the electronic medical records system and extracted using CRIS, thereby preserving patient anonymity.

### Potential risk factors

All potential risk factors were obtained at the time of the census. Several factors were extracted directly from structured fields within electronic medical records, including: sex, ethnicity, date-of-birth, and admission/discharge dates (used to calculate length of stay for the current SLaM forensic episode which was subsequently recoded into a binary variable: ≤ 18 months vs. > 18 months). Primary diagnosis was determined from the most recent Mental Health Review Tribunal report uploaded to the electronic medical records system and a binary variable was subsequently derived (psychotic disorder vs. other disorder). HCR-20 assessments [14] are regularly completed in SLaM forensic services; total scores on the most recent assessment were obtained where available.

Census forms were completed by the multidisciplinary clinical teams and used to capture a broad range of potential risk factors, including: current leave status, Mental Health Act section (collapsed to civil vs. forensic), recent episode of acute illness (psychosis, mania, or depression), current medication, concerns regarding medication compliance, and engagement in psychological therapy. Census forms included three items to determine the presence of learning disability, personality disorder (any), and psychopathy based on previously-completed assessments (if available) in the patient’s clinical record (i.e., assessments typically completed by forensic psychiatrists and psychologists as part of routine clinical care). For each diagnosis, clinical teams were asked whether the diagnosis was (i) not present, (ii) likely to be present but not formally confirmed (i.e., the clinical team suspect diagnosis is present but there are no recent/relevant assessments available in the patient’s clinical record to confirm this), or (iii) definitely present as confirmed using a validated assessment tool. Validated tools to assess personality disorder included the Minnesota Multiphasic Personality Inventory (MMPI [19]) and the Structured Clinical Interview for DSM-IV Axis II Personality Disorders (SCID-II [20]), and for psychopathy, included the Psychopathic Checklist Revised (PCL-R [21]) and Psychopathic Checklist Screening Version (PCL-SV [22]). We initially undertook analyses at all three levels (not present, likely present, and definitely present) for all three variables; however, for learning disability and personality disorder, statistical power was improved by collapsing across levels and no information was lost in doing so. These two variables were therefore subsequently collapsed to form binary variables (not present vs. likely or definitely present). Psychopathy was retained as a three-parameter variable owing to the fact that the association with absconsion was not consistent across the probable and definite diagnostic groups (see Table 2). Clinical teams additionally provided information on the current index offence, categorised as violent (e.g., assault, grievous/actual bodily harm, murder/manslaughter), acquisitive, drug, sexual, and other offences. These data were subsequently collapsed to violent vs. other; as it was not possible to determine whether sexual index offences were violent in nature, sexual offences were included in the ‘other’ category along with acquisitive, drug, and other offences. Clinical teams also indicated whether the patient had a history of sexual offending and whether the patient had previously absconded. The census form was also used to determine whether the patient had used substances in an inpatient setting and whether the patient had exhibited aggression as an inpatient (none; verbal aggression only; actual or attempted physical violence); both items were rated for the 12-month period prior to census completion.

**Table 2:** Logistic regression analyses examining demographic and clinical predictors of absconsion.

Risk factor	Absconders (*n* = 27)		Non-absconders (*n* = 108)		OR	(95% CI)	*p*
**Demographic factors**							
Age (years): mean (SD)	35.6	(11.5)	39.2	(11.7)	0.97	(0.93 to 1.01)	0.15
Female sex: *n* (%)	11	(10)	3	(11)	1.10	(0.29 to 4.26)	0.89
*Ethnicity*: *n* (%)							
White	7	(26)	26	(26)	(ref)	——-	——
Black African / Caribbean	12	(44)	34	(33)	1.31	(0.45 to 3.80)	0.62
Other	8	(30)	42	(41)	0.71	(0.23 to 2.18)	0.55
Length of stay > 18 months	12	(44)	61	(57)	0.62	(0.26 to 1.44)	0.26
*Leave status*: *n* (%)							
No leave / ground leave	11	(46)	44	(41)	(ref)	——-	——
Escorted community leave	6	(25)	28	(26)	0.86	(0.28 to 2.58)	0.78
Unescorted community leave	7	(29)	35	(33)	0.80	(0.28 to 2.28)	0.68
Forensic MHA section: *n* (%)	20	(80)	83	(79)	1.06	(0.36 to 3.14)	0.92
**Clinical factors**							
Psychotic disorder (primary): *n* (%)	22	(82)	83	(77)	1.33	(0.46 to 3.86)	0.61
Learning disability: *n* (%)	5	(21)	15	(14)	1.60	(0.52 to 4.92)	0.42
Recent episode acute illness: *n* (%)	10	(42)	39	(36)	1.25	(0.51 to 3.07)	0.63
Personality disorder (any): *n* (%)	14	(56)	53	(50)	1.27	(0.53 to 3.06)	0.59
*Psychopathy*: *n* (%)							
Not present	17	(71)	84	(79)	(ref)	——-	——
Likely (not confirmed)	7	(29)	10	(9)	3.41	(0.96 to 11.67)	0.06
Definite (formally assessed)	0	(0)	13	(12)	0.28	(0.00 to 1.82)	0.22

Note. LoS: Length of stay for current episode; MHA: Mental Health Act; OR: odds ratio. Missing data: ethnicity (*n* = 6); leave status (*n* = 4); MHA section (*n* = 5); learning disability (*n* = 5); recent episode of acute illness; (*n* = 4); personality disorder (*n* = 4); psychopathy (*n* = 4).

### Outcome

In January 2014 (approximately 26 months after initial census completion), incidents of absconsion were ascertained for all patients in the census cohort using CRIS. Specifically, CRIS was used to perform free-text searches of ‘events’ and ‘ward progress notes’ (i.e., entries made by clinical teams at multiple times throughout the day) within electronic medical records. The following search terms were used to identify entries containing any of the following key words: ‘abscon*’, ‘escap*’, ‘fail* to return’, and ‘AWOL’ (i.e., absence without official leave); each retrieved entry was then manually cleaned to identify those relating to genuine incidents of absconsion. Incidents were categorised into three types, (i) absconsion (i.e., running away from a member of staff, or refusing to return to the unit with a member of staff, whilst on escorted leave), (ii) escape (i.e., a successful or unsuccessful attempt to escape from the perimeter of the hospital), and (iii) failure to return from unescorted leave. In order to increase statistical power, these events were then subsequently collapsed to create a binary variable (absconsion vs. no absconsion) for each patient. In sensitivity analyses, we additionally examined the ability of the final weighted risk score to predict each of the three outcomes separately.

### Data analyses

All analyses were performed using Stata version 12. Univariable logistic regression analyses were initially performed to identify factors that distinguished patients who had absconded at least once during the follow-up period from those with had not. In order to derive a parsimonious regression model, factors that were significant predictors of absconsion in univariable analyses (*p* < 0.05) were then entered into a backward stepwise logistic regression model using a probability of 0.10 for variable entry and removal. Multicollinearity was assessed via the correlation matrix and indicated no issues with multicollinearity (*r*<0.30 for all correlations between the four independent variables retained in the final model). Factors retained in the multivariable model were then weighted according to their ability to discriminate between absconders and non-absconders using a methodology employed in the development of the Violence Risk Appraisal Guide (VRAG) [23]. Specifically, for each risk factor included in the final model, the total sample was stratified by the presence of that risk factor and absconsion rates were calculated within each stratum (absent vs. present). For each stratum, a weighting of one was then assigned for every full 5% plus or minus the base rate of absconsion in the total sample. A total score was derived for each participant by summing weighted scores for each risk factor included in the final model.

Several measures were used to assess the predictive accuracy of the final risk assessment scale. A receiver operator characteristic (ROC) analysis was first performed on the total scores, thus, for each possible score of the scale the true positive rate was plotted against the false positive rate. The area under the curve (AUC) statistic was subsequently derived, indicating the probability that a randomly selected absconder would obtain a higher risk classification on the risk scale than a randomly selected non-absconder [15]. The ROC analysis was also used to determine an optimal cut-off point on the risk assessment scale, that is, a cut-off yielding the best trade-off between sensitivity (the proportion of absconders correctly classified as high-risk) and specificity (the proportion of non-absconders correctly classified as low-risk). The positive (PPV) and negative predictive values (NPV) were subsequently computed at the optimal cut-off, indicating the probability that a high-risk patient will abscond and that a low-risk patient will not abscond, respectively. Consistent with Fazel and colleagues [24,25], we also computed the diagnostic odds ratio, defined as the odds that an absconder will be classified as a being at high-risk relative to the odds that a non-absconder will be classified at high-risk.

## Results

### Sample demographics and incidents of absconsion

Across the total sample (*N* = 135), the mean age at study commencement was 38.5 years and the majority of patients were male (90%). Psychotic disorder was the most common primary diagnosis (78%) while just over half of the sample had a primary or co-morbid diagnosis of a personality disorder (51%). At the time of census completion, the median length of stay in forensic inpatient wards for the total sample was 406 days (range: 7–2510) and approximately one third of the cohort had been granted unescorted community leave (32%).

Absconsion data were obtained at the two-year follow-up for all patients examined in the 2011 census. In total, 27 patients (20%) absconded from a forensic ward on at least one occasion during the follow-up period; between them, these patients were involved in 56 separate incidents of absconsion with a median of one incident per patient (range: 1–8). Incidents were most commonly classified as failures to return to the unit (36 incidents committed by 14 patients), followed by escapes (14 incidents committed by 7 patients), whilst relatively few related to incidents of absconsion whilst on escorted leave (6 incidents committed by 3 patients). Given that only one of the nine forensic wards was a low-secure ward, incidents in low-security appeared to be somewhat over-represented (21 incidents vs. 35 in medium-security); of note, the majority of escapes were from low-secure wards.

### Univariable associations between potential risk factors and absconsion

Univariable logistic regression analyses were conducted to examine the association between potential risk factors and absconsion. As shown in Table 2, whilst absconders were somewhat younger in age relative to non-absconders (mean age: 35.6 vs. 39.2 years), this association did not achieve statistical significance (OR = 0.97, *p =* 0.15). Indeed, none of the demographic factors (i.e., sex, ethnicity, length of stay, leave status, and forensic MHA section) were significant predictors of absconsion (*p* > 0.05 for all factors). Of the clinical factors examined (Table 2), absconsion was not significantly associated with a primary diagnosis of psychotic disorder, having a definite or probable diagnosis of learning disability, a recent episode of acute illness, or personality disorder (*p* > 0.05). Relative to those without psychopathy, the odds of absconsion were higher among those patients who were noted by the clinical teams to have a likely diagnosis of psychopathy but who had not formally been assessed (OR = 3.41). In contrast, those definitely meeting criteria for psychopathy (assessed using the PCL-R or PCL-SV) were less likely to abscond (OR = 0.28); however, neither parameter achieved statistical significance (*p* > 0.05).

Treatment-related factors and their association with absconsion are presented in Table 3. Neither medication non-compliance, clozapine treatment, nor engagement in psychological therapy were significant predictors of absconsion (*p* > 0.05). In contrast, absconsion was significantly associated with a number of offending/behavioural factors. Patients who absconded were more likely to have an ‘other’ index offence (OR = 3.00, *p =* 0.02), a history of sexual offending (OR = 2.62, *p =* 0.04), and to have absconded previously (OR = 2.60, *p =* 0.04). With regards to inpatient behaviour in the twelve months prior to census completion, those who had engaged in substance use were significantly more likely to abscond (OR = 3.93, *p =* 0.01). Furthermore, relative to patients who exhibited no aggression, the odds of absconsion were approximately four times higher among patients who had been verbally aggressive (OR = 3.93, *p =* 0.01), but were lower among patients who had been physically violent (OR = 0.69, *p =* 0.56).

**Table 3:** Logistic regression analyses examining treatment-related and offending/behavioural predictors of absconsion.

Risk factor	Absconders (*n* = 27)		Non-absconders (*n* = 108)		OR	(95% CI)	*p*
**Treatment-related factors**							
Medication non-compliance; *n* (%)	15	(63)	55	(51)	1.58	(0.64 to 3.91)	0.33
Clozapine treatment; *n* (%)	5	(20)	25	(24)	0.80	(0.27 to 2.35)	0.69
*Psychological therapy; n* (%)							
Engaged (good attendance)	9	(39)	42	(40)	(ref)	——-	——
Refused or poor attendance	11	(48)	43	(41)	1.19	(0.45 to 3.18)	0.72
Not offered or unable to attend	3	(13)	20	(19)	0.70	(0.17 to 2.87)	0.62
**Offending and behavioural factors**							
HCR-20 total score; mean (SD)	25.6	(5.3)	25.1	(5.6)	1.02	(0.94 to 1.11)	0.67
Other index offence; *n* (%)	15	(60)	36	(33)	**3.00**	**(1.23 to 7.34)**	**0.02**
History of sexual offending; *n* (%)	13	(54)	32	(31)	**2.62**	**(1.06 to 6.48)**	**0.04**
Previous absconsion; *n* (%)	12	(50)	30	(28)	**2.60**	**(1.05 to 6.42)**	**0.04**
Inpatient substance use; *n* (%)	12	(48)	25	(24)	**2.99**	**(1.21 to 7.38)**	**0.02**
*Inpatient aggression*; *n* (%)							
None	10	(42)	59	(55)	(ref)	——-	——
Verbal aggression	10	(42)	15	(14)	**3.93**	**(1.39 to 11.17)**	**0.01**
Physical violence	4	(16)	34	(31)	0.69	(0.20 to 2.38)	0.56

Note. HCR-20: Historical Clinical Risk-Management—20; OR: odds ratio. Missing data: medication non-compliance (*n* = 4); clozapine treatment (*n* = 5); psychological therapy (*n* = 7); HCR-20 (*n* = 22); other index offence (*n* = 2); history of sexual offending (*n* = 8); previous absconsion (*n* = 3); inpatient substance use (*n* = 4); inpatient aggression (*n* = 3).

### Multivariable model and scale weighting

A stepwise logistic regression analysis was performed on the five factors that achieved statistical significance in univariable analyses, namely, other index offence, history of sexual offending, previous absconsion, inpatient substance use, and inpatient aggression (note, dummy variables coding for verbal aggression and physical violence were both included in the multivariable model). Physical violence and other index offence did not improve overall prediction and were therefore excluded from the model in Step 1 and Step 2, respectively. The four remaining items were then weighted according to their ability to discriminate absconders from non-absconders using the method described above; briefly, after stratifying by each risk factor, a weight of one was assigned for every full 5% ± the base rate of absconsion in the total sample. For example, the rate of absconsion among those with and without a history of sexual offending was 29% and 13%, respectively; as these rates are a full 5% plus and minus the absconsion rate in the total sample (20%) a history of sexual offending received a weight of plus one whilst the absence of a history of sexual offending was given a weight of minus one. As shown in Table 4, using this procedure, a history of sexual offending and previous absconsion were assigned the same weighting whilst inpatient substance use and verbal aggression were weighted more heavily. Possible minimum and maximum scores on the weighted scale ranged from minus four to eight.

**Table 4:** Weighting for individual scale items.

	Risk factor absent		Risk factor present	
Risk factor	Absconsion rate	Weighted score	Absconsion rate	Weighted score
History of sexual offending	13%	- 1	29%	+ 1
Previous absconsion	13%	- 1	29%	+ 1
Inpatient substance use	14%	- 1	32%	+ 2
Inpatient verbal aggression	13%	- 1	40%	+ 4
Total score	Minimum	- 4	Maximum	+ 8

### Predictive accuracy of the weighted scale

**Fig 1:**
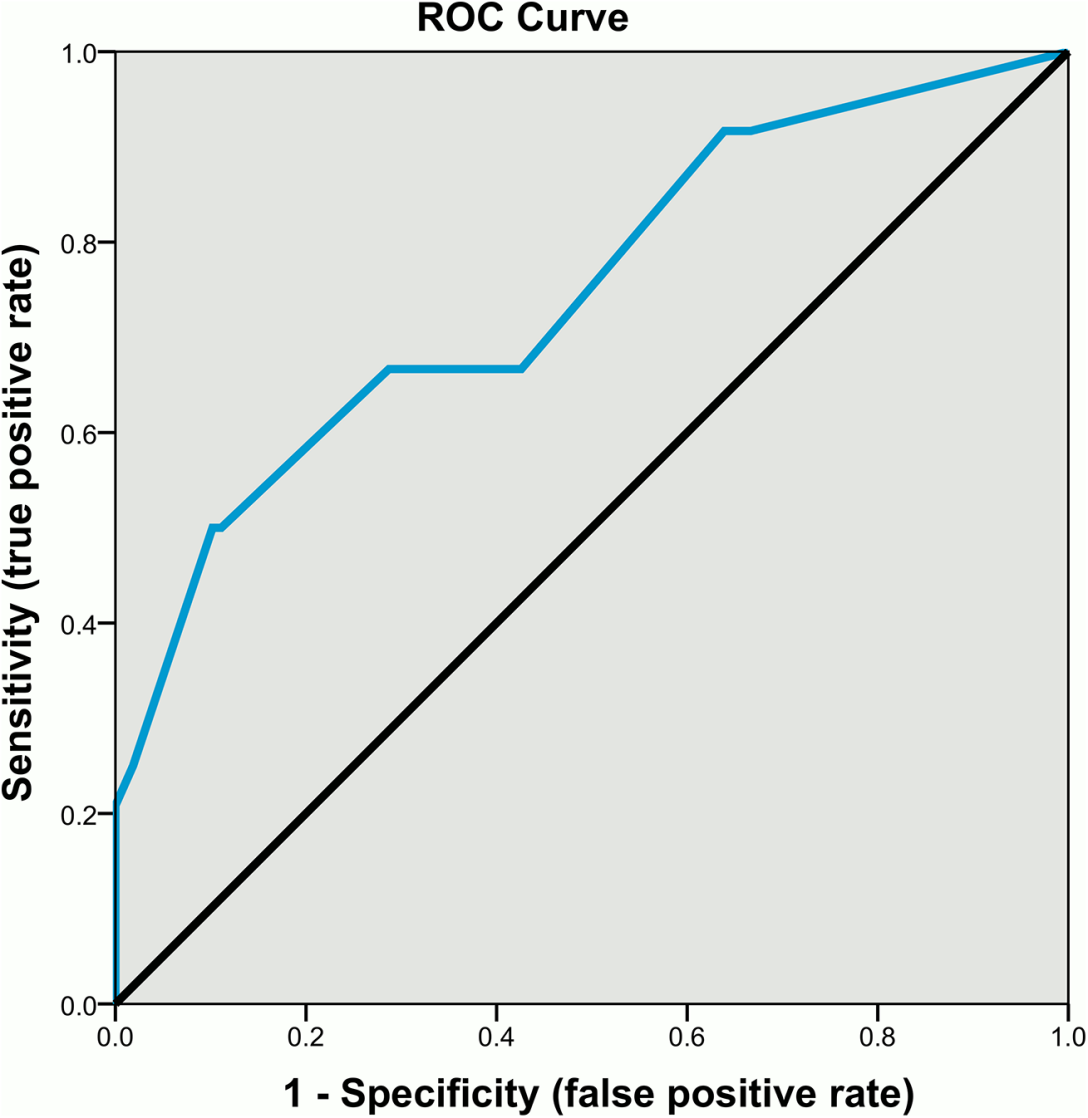
Receiver operator characteristic (ROC) curve for total scores on the four-item weighted absconsion risk scale (AUC = 0.75).

ROC analysis was performed on the total scores of the weighted scale to examine the predictive accuracy of the final risk assessment scale and identify a suitable cut-off score. These analyses (Fig 1) yielded an AUC statistic of 0.75 (95% CI = 0.63 to 0.87) and determined that a cut-off score of one (i.e., ≤ zero = low-risk; ≥ one = high-risk) represented the best trade-off between sensitivity and specificity. Applying this cut-off, 36% and 64% of the sample were classified as high- and low-risk, respectively. The proportion of absconders correctly classified as high-risk (sensitivity) and the proportion of non-absconders classified as low-risk (specificity) was 0.67 (95% CI = 0.45 to 0.84) and 0.71 (95% CI = 0.62 to 0.79). The probability that a patient classified as high-risk would go on to abscond was low (PPV = 0.34; 95% CI = 0.21 to 0.49), however, the likelihood that a low-risk patient would not abscond was high (NPV = 0.91; 95% CI = 0.82 to 0.96). Finally, the diagnostic OR indicated that the odds of being classified as high-risk were approximately five times higher among absconders relative to non-absconders (OR = 4.97; 95% CI = 1.93 to 12.79).

### Analyses by incident subtype

Sensitivity analyses were conducted to determine whether total scores on the weighted risk scale predicted each of the three incident subtypes (i.e., failure to return, escape, and absconsion). Logistic regression analyses indicated that scores on the weighted risk scale were significantly associated with each of the outcome incident subtypes (*p* < 0.05 for all). Specifically, for every one point increase on the weighted risk scale, the odds of a patient failing to return increased by 1.21 fold (95% CI = 1.02 to 1.45), the odds of escaping or attempting to escape was increased by 1.50 fold (95% CI = 1.15 to 1.95) and the odds of absconsion increased by 1.46 fold (95% CI = 1.02 to 2.09).

## Discussion

This is the first truly prospective cohort study to examine factors predicting absconsion in a forensic psychiatric inpatient population. During the two-year follow-up, one in five patients absconded from medium- and low-secure inpatient settings (with incidents occurring in low-security appearing to be disproportionately high), these incidents were largely failures to return to the ward whilst on community leave. We found that the strongest predictors of absconsion were related to historical behaviour (a history of sexual offending and prior absconsion) and recent inpatient behaviour (substance use and verbal aggression occurring during the past twelve months). A weighted scale based these four items was found to have adequate predictive accuracy as indicated by moderate-to-high AUC, sensitivity, and specificity values; however, while the NPV was high, the PPV for the scale was very low. Such findings demonstrate that it is at least possible to develop a brief, empirically-derived absconsion risk assessment scale which could be trialled in forensic settings.

Our findings are broadly consistent with those of previous retrospective studies conducted in forensic inpatient populations. In the current study, having an index offence classified as ‘other’ (which included sexual offences) was a significant predictor of absconsion in univariable analyses, but was not retained in the final model after a history of sexual offending was included. In contrast, three previous studies reported that a sexual index offence was not significantly associated with absconsion [5,7,10]; yet Beer and colleagues found that a history of sexually inappropriate behaviour was associated with absconsion at the trend level [13]. Together, these findings suggest that whilst sexual offending may be an important risk factor for absconsion, index offence alone may not be particularly informative; thus, taking into consideration any history of sexual offending may improve prediction of absconsion. Our finding that absconsion was associated with a history of previous absconsion was unsurprising and is consistent with all four previous studies that have included prior absconsion (actual or attempted) as a potential risk factor [5,6,10,13]. However, the practical implications of identifying past behaviour as a risk factor for future behaviour are limited as such behaviours cannot be targeted with interventions. A novel finding from our study was that recent inpatient behaviours, namely, substance use and verbal aggression, were associated with absconsion; indeed these factors showed better ability to discriminate between absconders and non-absconders than historical factors. Whilst ours is the first study to have examined these specific inpatient behaviours, Brook and colleagues also reported that absconsion was associated with impulsive/aggressive behaviour on the ward [5]. Thus, it may be that recent behaviour provides a good indication of current levels of impulsivity which might in turn contribute to absconding behaviour.

In contrast with some previous forensic studies, but not all, absconsion was not predicted by any of the demographic factors examined, although this may reflect a lack of statistical power. Whilst some studies have reported that younger age [5,9,26], male sex [6], and ethnicity [6] are associated with absconsion, others have not [5,6,7,9,10,13,26,27]. Whether such factors predict absconsion may depend on the demography of the sample and the clinical setting. Indeed, studies conducted in general psychiatric settings have yielded more consistent findings than forensic studies [11], and have typically shown that absconders are more likely to be younger in age [28,29,30,31,32,33]. A similar consideration may apply in regards to clinical factors; previous studies in acute settings have observed higher rates of absconsion among patients with schizophrenia [28,30,31,33], but absconsion was not predicted by a diagnosis of psychotic disorder in the current study or in previous forensic studies [5,8,10,27]. Thus, psychotic diagnoses may be of greater relevance in acute general psychiatry settings, possibly reflecting the fact that acutely unwell patients in forensic settings will likely be given less leave and will therefore have less opportunity to abscond. However, as noted above, whether a potential risk factor can be found to predict an outcome depends on the prevalence (or variability) of the exposure in the sample; as the majority of patients in our study had a primary diagnosis of psychotic disorder, it is possible that we did not have sufficient numbers of non-psychotic patients to allow us to determine whether psychotic disorder is indeed a risk factor for absconsion.

Given the findings of previous studies conducted in both forensic settings [7,11,26] and general psychiatric hospitals [34,35,36], it was surprising that neither psychopathy nor personality disorder predicted absconsion. Interestingly, we found that whilst those with a likely diagnosis of psychopathy were three times more likely to abscond than those without psychopathy, none of the patients with a definite diagnosis absconded. It may be that patients who were deemed to require a formal assessment of psychopathy and who were known to fulfil psychopathy criteria were placed under more restrictive leave conditions, thereby reducing the risk of absconsion. However, those without a ‘definite’ formal diagnosis (but who might have some traits) might not be subject to the same restrictions. Consistent with this hypothesis, we also found that patients who had exhibited recent physical violence in an inpatient setting were less likely to abscond than those who had not. Thus, patients deemed at particularly high-risk (i.e., those with known psychopathy and those who have been physically violent) may be at lower risk of absconding because they have fewer opportunities to do so or because their risk management plans address a comprehensive range of risk scenarios.

An alternative explanation for the lack of association between ‘definite psychopathy’ and absconsion relates to the fact that psychopathy ratings were made by the clinical teams using previously-collected data (i.e., PCL-R or PCL-SV assessments not completed for the purposes of this study). Thus, these assessments were carried out by different clinicians with varying skill levels. Whilst it is dictated within our NHS trust that all forensic psychiatrists and psychologists must undergo formal PCL-R/PCL-SV training before carrying out these assessments, we were not able to examine the reliability of assessments across raters. However, as assessments were typically completed by the multidisciplinary team (rather than individual clinicians) the reliability and validity of these assessments are likely to be high. Finally, in contrast to hypotheses and two previous forensic studies [5,13], none of the treatment-related factors we examined were significantly associated with absconsion.

A secondary aim of the study was to assess the feasibility of developing a brief absconsion risk scale. The sensitivity and specificity values (0.67 and 0.71, respectively) indicated that the scale had moderate ability to classify absconders as high-risk and non-absconders as low-risk. One major limitation was that the probability that a patient identified as being at high-risk for absconsion would actually abscond was low (PPV = 0.37). However the negative predictive value was high (NPV = 0.91). These findings indicate that the scale over-identifies those who might be at high-risk of absconding, but that the measure can classify low-risk patients with high levels of accuracy. Whilst this raises questions about the extent to which this scale can be practically applied in a forensic setting, it is important to note that these values are very similar to those reported for well-established violence risk assessment scales. In a recent meta-analysis, Fazel and colleagues reported sensitivity and specificity values of 0.92 and 0.36, respectively, and positive and negative predictive values of 0.41 and 0.91 for the ability of these tools to predict violence [24]. Thus, the predictive accuracy of our absconsion tool is similar to that reported for widely-used violence risk assessment measures.

One important consideration when evaluating the predictive accuracy of the weighted risk scale is the observational (i.e., naturalistic) nature of this study which likely affected the findings. Not all patients included in the study had the same opportunity to abscond over the study period, and this in turn is likely to be related to the risk of absconsion. For example, by definition, patients in the low-secure ward were placed under less restrictive conditions and therefore likely had greater opportunity to abscond. However, these patients were likely to be at lower risk of absconsion and other adverse outcomes (i.e., in order to deem them suitable for treatment in a low-secure environment); thus, potentially reducing the association between true risk factors (e.g., psychopathy and physical violence) and absconsion. Even within a medium-secure setting, clinical interventions may have had an effect on the observed findings. It is highly possible that clinical teams may have imposed additional restrictions on those at high-risk for absconsion, or those who were acutely unwell, which may have prevented this outcome occurring.

### Implications

The clinical utility and acceptability of using an absconsion risk screening tool with the PPV and NPV of our instrument will depend on the clinical and security responses that are elicited by high and low scores. If such a scale is used to classify high-risk individuals for the purposes of identifying those who might benefit from a relatively benign psychological or supervisory intervention then use of the tool may have some merit. However, in view of the poor PPV value, we suggest that it would be inappropriate to infer that a high score should be the sole basis for decisions about a patient’s security needs. On the other hand, it may be possible to use the tool to screen out those at very low risk of absconding who might be eligible for less restrictive leave conditions. Similarly, a study investigating the predictive validity of a newly-developed violence risk assessment measure, demonstrating similar psychometric properties to the absconsion risk scale developed in the current study, proposed that such an instrument could be utilised to screen out patients at very low risk of violence prior to completing more detailed assessments [25]. Owing to the paucity of rigorously conducted studies in this area, decisions about access to leave (which in the UK are made by clinical teams and the Ministry of Justice) are not informed by absconsion-specific studies. Instead, decisions are based on professional judgement which is likely influenced by a range of generic risk factors for other outcomes (e.g., violence) and/or by taking a generally and generically cautious approach to leave for all patients irrespective of specific risk factors. Both of these approaches may mean that individuals who are actually at low-risk for absconsion are given more restrictive leave conditions than are actually warranted, using absconsion-specific risk tools to identify those at low-risk for this outcome may help to reduce this possibility.

Despite the fact that our scale may not accurately identify those at high-risk of absconsion, the results of the current study are clinically useful in that they provide suggestions for interventions which may help to reduce the risk among forensic psychiatric patients. Two indices of recent inpatient behaviour, verbal aggression and substance use, were found to be the strongest predictors of absconsion. Previous work by our group has indicated that cognitive-skills programmes targeting social problem-solving skills and thinking styles can be effective in reducing these behaviours in forensic populations. Specifically, in a randomised controlled trial of the Reasoning and Rehabilitation (R&R) programme, we observed that forensic psychiatric patients randomised to R&R showed reductions in verbal aggression relative to those receiving treatment as usual and that this effect was maintained at 12-months post-treatment [37]. Patients who completed the full R&R programme additionally demonstrated significant reductions in substance use. The current findings suggest that reductions in these behaviours might be associated with a reduction in absconsion; indeed, we also observed a significant effect of R&R on the number of leave violations (which included absconsion incidents) with stronger effects among those completed treatment [37]. Thus, programmes such as R&R may help to reduce the risk of absconsion and other impulsive/antisocial inpatient behaviours by promoting adaptive thinking styles.

### Limitations

A major limitation of the current study is the small sample which limited our ability to identify significant predictors of absconsion (i.e., due to a lack of statistical power) and also causes problems for model over-fitting in multivariable analyses. Statistical power was further reduced by the fact that the study suffered from missing data (particularly problematic for HCR-20 scores); however, only three patients were missing data on variables that were included in the final weighted risk scale. Furthermore, in this small sample we were not fully able to examine different types of absconding behaviour separately. Whilst we were able to perform sensitivity analyses to confirm that scores on the final weighted risk scale significantly predicted each of the three outcome incident subtypes (failure to return, escape, and absconsion), the sample size was not sufficiently large for us to be able to perform multivariate analyses to identify factors associated with each of the outcomes. Given that these acts are heterogeneous in nature, it is possible that these behaviours are associated with different risk factors. A further limitation relates to the extent to which our findings can be generalised to other populations given that the study was conducted in a single NHS trust. However, our results are largely consistent with previous retrospective studies in forensic populations, thereby increasing our confidence that such factors are relevant to other samples. The use of routinely-collected data (i.e., not obtained for the purposes of this study) is a potential limitation. This is particularly problematic for ratings of comorbid psychiatric conditions such as personality disorder and psychopathy where there was a degree of uncertainty regarding the extent to which a patient met diagnostic criteria. These diagnoses were rated as ‘not present’, ‘possibly present but not formally assessed’, or ‘definitely present as determined based on a formal assessment’ depending on whether a recent/relevant assessment, using a validated tool, was available in the patient’s clinical record. This uncertainty may have contributed to the lack of association between these diagnoses and absconsion. However, the use of data collected by the treating team (as opposed to research clinicians) adds to the external validity of the study as the risk scale is scored using ‘real world’ data and the lack of definite information on personality disorder and psychopathy reflects the realities that many clinical teams face. One major limitation is that we were not able to perform cross-validation analyses of the risk assessment tool in an independent sample, thus, these findings should be interpreted with caution. Finally, as discussed above, one issue that is common to all observational predictive studies, is that one cannot control for the effect of interventions that may be implemented to reduce the risk of absconsion in specific high-risk groups. For example, the fact that definite psychopathy and physical violence were found to be negatively associated with absconsion may reflect the fact that patients with these characteristics had more restrictive leave conditions and therefore had fewer opportunities to abscond.

## Conclusions

This preliminary study represents the first attempt to conduct a truly prospective investigation of absconsion in a forensic psychiatric inpatient setting. Our findings suggest that absconding behaviour among forensic psychiatric inpatients is most strongly associated with recent inpatient behaviours that may be indicative of impulsive, rule-breaking, and antisocial traits. Psychological treatments aimed at promoting adaptive thinking styles may help to reduce these traits and the risk of absconsion. We additionally attempted to develop an empirically-derived scale to assess risk of absconsion in forensic settings; this scale had poor predictive ability but showed potential for identifying those at low-risk for absconsion. Whilst we cannot recommend that this tool is used in clinical practice without further external validation in an independent sample, we have at least shown that it is possible to develop a brief risk assessment tool and we hope this stimulates further research in this area.
